# Measuring stem cell dynamics in the human colon – where there’s a wiggle, there’s a way†

**DOI:** 10.1002/path.4422

**Published:** 2014-10-10

**Authors:** Simon J Leedham

**Affiliations:** Gastrointestinal Stem Cell Biology Laboratory, Wellcome Trust Centre for Human Genetics, Roosevelt Drive, University of OxfordOxford, OX3 7BN, UK

**Keywords:** stem cell, intestine, mtDNA, crypt map, cytochome c oxidase, FAP

## Abstract

The last decade has seen huge improvements in our understanding of intestinal stem cell biology, with major advances arising from the ability to transgenically label, and thus identify, murine stem cells and their progeny. In the human, transgenic labelling is not an available option and stem cell dynamic observations have been based on rare hereditary mutations and polymorphisms. Somatic mitochondrial DNA mutations cause a histochemically detectable, but neutrally selected, change in cytochrome *c* oxidase (CCO) enzyme activity and when this occurs in an intestinal stem cell, it can be used as an effective clonal marker in both health and disease. The intestinal crypt is the functional unit of the gut. Daughter cells are ‘born’ in the stem cell niche at the crypt base and proliferate, differentiate, and then apoptose as they migrate along the vertical crypt axis over 5–7 days. This stereotypical architecture provides a historical record of cell dynamics, as the distance travelled along the crypt axis is proportional to the time since the daughter cell was born. By staining, identifying, and carefully reconstructing crypt maps from serial *en face* sections of partially mutated mtDNA crypts, clonal ribbon images can be generated. ‘Wiggles’ in the width of the clonal ribbon reflect mtDNA mutated stem cell expansion or contraction events and these biological observations are applied in mathematical models. This clever approach is able to infer temporal evolutionary dynamics from a static, single time point measurement, in both normal and familial adenomatous polyposis tissue. As we have seen in the mouse, the simple ability to identify stem cell progeny can lead to a vast expansion in our understanding of stem cell evolution. The use of these techniques to trace recent stem cell dynamics in the human colon makes some headway into the knowledge gap in our understanding of murine and human intestinal stem cell biology.

The mammalian intestine is lined by a single layer of epithelium that undergoes continual replacement every 5–7 days. This remarkable turnover is supported by adult intestinal stem cells located in the base of flask-shaped invaginations called crypts – the basic functional units of the gut. In the small intestine, several crypts contribute epithelium to finger-like projections called villi. Stem cells are defined functionally, firstly by their ability to self-renew and secondly by multipotency – the capacity to produce daughter cells that undergo limited lineage differentiation to produce all of the post-mitotic specialized cells of the gut. Intestinal epithelial daughter cells originate in the base of the crypt and migrate along the vertical crypt–villus axis, becoming progressively more differentiated as they age. Spontaneous apoptosis with subsequent shedding or phagocytosis occurs at the luminal surface about a week after daughter cells are ‘born’. Maintenance of stem cells, daughter cell proliferation, and induction of differentiation (cell fate) are rigidly controlled by interacting gradients of intrinsic epithelial and extrinsic mesenchymally derived morphogen signalling pathways 1.

The last decade has seen tremendous advances in our understanding of the dynamics of this complex tissue organizational hierarchy. The major advance has been the ability to fluorescently label cell populations in transgenic mice. The simple ability to identify stem cell progeny has allowed real biological measurements to underpin mathematical modelling of stem cell dynamics. Further advances have come from the identification and fluorescent labelling of genes selectively expressed by stem cells. Lineage tracing experiments have been used to demonstrate long-term self-renewal and multipotency criteria to validate genes as *bona fide* stem cell markers. Landmark achievements and advances in understanding murine stem cell dynamics have rapidly followed. We now know that murine intestinal stem cells rarely divide asymmetrically, as previously believed, but instead follow a pattern of neutral drift, with clonal expansion and contraction occurring in perfect balance in intestinal homeostasis 2,3. Furthermore, stemness is principally not an intrinsic cell-defined property; instead, it appears to be determined by proximity to contextual cues from the stem cell niche. A spectrum of stem-cell competence exists with variable bias towards self-renewal or differentiation dependent on distance from a ‘sweet spot’ in the crypt base 4. Consistent with this spectrum, quiescent or reserve stem cell populations 5, and different secretory precursor cells that have ostensibly exited the niche, have the ability to reactivate stem cell potential at times of need and regenerate the crypt when damaged 6,7.

Our understanding of murine intestinal stem cell dynamics has thus expanded exponentially, but what about human stem cells? Clearly the use of transgenic lineage tracing technology cannot be applied and stem cell dynamic observations in the human have been based on rare hereditary changes such as X-inactivation in G6PD heterozygotes 8, polymorphisms in the gene coding for the enzyme *O*-acetyltransferase 9, and an isolated patient with XO/XY chimerism 10. More recently, a technique that capitalizes on the high somatic mutation rate of mitochondrial DNA has been successfully utilized to clonally mark human intestinal tissue 11–;14. Stochastic mitochondrial DNA (mtDNA) somatic mutation causes a histochemically detectable change in cytochrome *c* oxidase (CCO) enzyme activity and when this occurs in an intestinal stem cell, the cell lineage can be traced histochemically using a blue stain (CCO−) against a brown (CCO+) background. Somatic mtDNA mutation increases with age, can be detected in both normal and adenomatous crypts, and remarkably appears to exert no significant positive or negative selection pressure on affected cells 15,16. Writing in *Cell Reports*, Baker *et al* have optimized the use of this technique, cleverly exploiting the stereotypic architecture of the crypt to provide the biological measurements necessary to undertake plausible mathematical modelling of *in vivo* human intestinal stem cell dynamics 17. By meticulously examining serial, *en face* sections of partially mutated crypts, they reconstruct a crypt map to show a ribbon of mutated cells as they migrate along the vertical axis of the intestine, like a plume of smoke emerging from a lit match (Figure 1A). Recognizing that the distance travelled along this crypt axis is proportional to the time since the daughter cells were born in the crypt base, the authors have identified a historical record reflecting past events in the stem cell pool over the 5–7 days it takes daughter cells to migrate. Expansion of the ribbon corresponds to expansion of the mutant cell pool, whereas ribbon contraction reflects lineage death and these are recorded as ‘wiggles’ in the ribbon width. Clonal extinction can also be momentarily detected as a terminal ribbon disconnected from the crypt base, just as a rising smoke plume can be temporarily seen after a match is extinguished (Figure 1B). By analysing the size and distribution of changes in ribbon width, the authors determine that the crypt base contains a small number of functional stem cells (approximately 6) that predominantly divide symmetrically with balanced clone expansion and contraction, resulting in a neutral drift homeostatic clonal evolution pattern. Importantly, by examining both normal and early adenomatous lesions in attenuated and full-blown familial adenomatous polyposis (FAP), they show a similar but more pronounced neutral drift pattern with an increase in functional stem cell number and loss/replacement rate, indicating more vigorous stem cell competition in these conditions.

**Figure 1:**
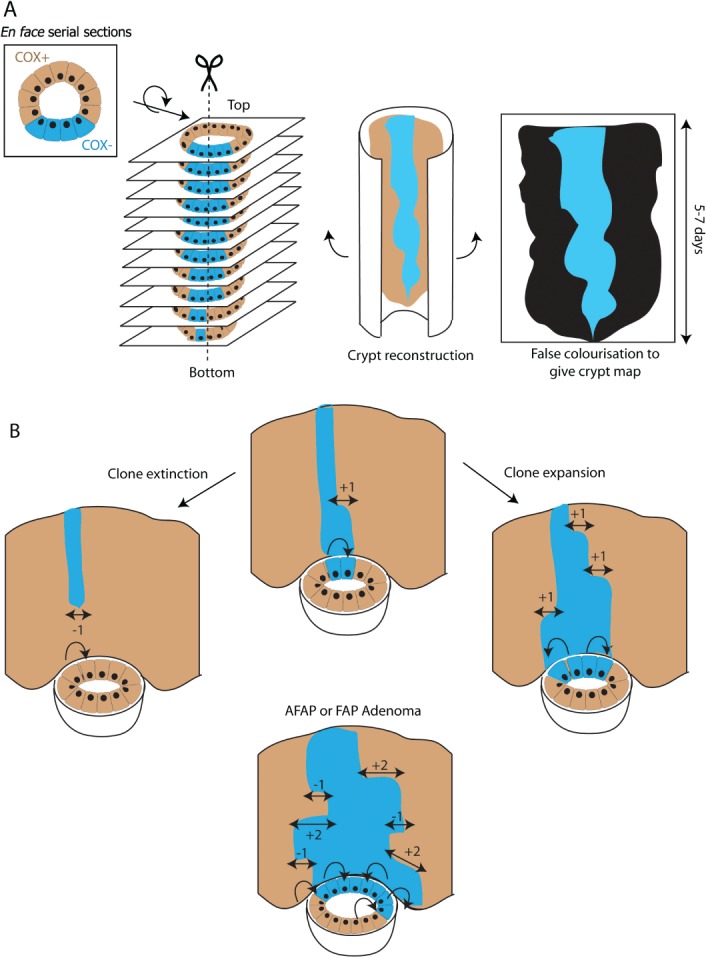
(A) Crypt reconstruction technique. Somatic mtDNA mutations cause a defect in cytochrome *c* oxidase (CCO) enzyme activity. Frozen colonic resection tissue is histochemically stained for CCO enzyme activity, with enzyme deficiency (CCO−) showing as a blue stain against a brown (CCO+) background. Partially mutant crypts are identified on aligned *en face* serial sections. Software processing generates a falsely coloured crypt map, with the longitudinal axis recording cell dynamics in the 5–7 days that it takes daughter cells to migrate from the bottom to the top of the crypt. (B) Clonal dynamics in the reconstructed crypt maps. CCO − clonal ribbons (blue) reflect dynamic changes in the stem cell niche. Clonal expansion occurs when a CCO − stem cell divides symmetrically at the expense of a CCO + cell (curved arrow), identified as +1 ‘wiggle’ in the clonal ribbon (double-headed arrow). Clonal extinction, resulting from division of a CCO + cell at the expense of a CCO − clone, can still be momentarily identified as a clonal ribbon disconnected from the crypt base, migrating up the crypt. Clonal contraction is measured as a −1 ‘wiggle’ in the ribbon. In an attenuated (AFAP) or full-blown familial adenomatous polyposis (FAP) microadenoma, there is an increase in stem cell number in the niche and more vigorous stem cell competition causes a greater number of ‘wiggles’ in the clonal ribbon.

By assessing mutant crypt patch sizes in normal tissue and non-dysplastic regions from AFAP and FAP resected colon, Baker *et al* have also measured the important, but often neglected, evolutionary dynamics of crypts themselves, as it is an increase in crypt fission that underpins the growth of most adenomas. Mathematical modelling shows that normal human crypts rarely divide, perhaps only once or twice in a lifetime, but that this rate is increased ten-fold in small adenomas. On the face of it, this increase isn’t as large as might be expected in an *APC*-deficient lesion, with an established greater number of stem cells per crypt. However, the lesions necessarily assessed here were small microadenomas to allow accurate reconstruction of the crypt architecture. It is plausible that fission increases as the adenoma grows and the crypt architecture becomes more distorted. This observation is consistent with the predicted slow growth rate of adenomas, which have been estimated to take more than 20 years to develop into a carcinoma 18.

The paper by Baker *et al* 17 is important because it utilizes the unique architecture of the intestine to infer temporal evolutionary dynamics from a static, single time point measurement. As we have seen in the mouse, the simple ability to identify stem cell progeny can lead to a vast expansion in our understanding of stem cell evolution and this paper makes some headway into the knowledge gap between human and murine intestinal stem cell dynamics. As with all mathematical modelling, the paper makes some assumptions of parameters such as stem cell number, cell cycle rate, speed of migration, and loss/replacement rate. Wherever possible, these assumptions are based on historical measurements 19,20 but it is a reflection of the paucity of our knowledge in this field that many of these measurements are more than 20 years old and of limited accuracy. Another assumption is that cell migration is measured as an ensemble average, with all cells assumed to move at the same speed along the length of the crypt and survive the same length of time. As some of the rarer specialized cell types such as enteroendocrine and tuft cells live considerably longer than enterocytes or goblet cells 21, this assumption is unlikely to be completely correct. Although the cell numbers involved are small, this does mean that the finer details of stem cell architecture and evolution could not be resolved in this study. Despite the meticulous nature of the serial sectioning here, it has still not been possible to identify the cell at the origin of the ribbon. This would have particular relevance for the apparent clonal extinction events (Figure 1B). As the accumulation of somatic mtDNA mutations can only occur in long-lived cells and the cell of origin of these clones is disconnected from the crypt base, it is assumed that mid-crypt ribbons are the migrating final progeny of a recently extinguished clone, rather than a true mid-crypt clonal founder. However, recent work in the mouse has indicated that a long-lived subpopulation of quiescent *Dckl1*+ tuft cells survive outside of the crypt base and can act as the cell of origin of cancer 21. It would be very interesting to see if any cells in the human share this ability. Perhaps the future combination of the mtDNA technique with immunohistochemistry for rarer intestinal lineages or *in situ* hybridization for established stem cell marker genes will help to further characterize these elusive clonal founder cells in the human.

Understanding stem cell dynamics is integral for our comprehension of basic neoplasia biology. Cancer is a disease of mutated stem cells and has historically been thought to originate from the crypt base, a paradigm supported by landmark stem cell marker-focused publications 22,23. One of the issues with stem cell marker-driven work is that there is a danger of defining a stem cell solely by marker expression rather than by characteristic functional parameters. This emphasis appears to be changing with the observation that a wider range of cells than previously believed can demonstrate stem cell function 4,6,7, given the appropriate spatiotemporal cues. Furthermore, the demonstration that murine intestinal cancers can arise from cells normally situated outside of the crypt base 21 or from dedifferentiated cells 24 in an altered mucosal microenvironment means that there is a pressing need to expand our knowledge of the cell of origin of human tumours. Baker *et al* have combined meticulous tissue processing with a simple histochemical stain to define a powerful technique for assessing stem cell dynamics *in vivo* in the human intestine. This toolkit will be used to help define stem cell dynamics in a range of human inflammatory and neoplastic conditions, as it is only by understanding the biology of tumours, including the cell of origin, can we hope to design drug treatments capable of tackling the dynamic stem cell environment in a human cancer.

## References

[b1] Scoville D, Sato T, He X (2008). Current view: intestinal stem cells and signaling. Gastroenterology.

[b2] Snippert HJ, van der Flier LG, Sato T (2010). Intestinal crypt homeostasis results from neutral competition between symmetrically dividing Lgr5 stem cells. Cell.

[b3] Lopez-Garcia C, Klein AM, Simons BD (2010). Intestinal stem cell replacement follows a pattern of neutral drift. Science.

[b4] Ritsma L, Ellenbroek SI, Zomer A (2014). Intestinal crypt homeostasis revealed at single-stem-cell level by *in vivo* live imaging. Nature.

[b5] Tian H, Biehs B, Warming S (2011). A reserve stem cell population in small intestine renders Lgr5-positive cells dispensable. Nature.

[b6] van Es JH, Sato T, van de Wetering M Dll1+ secretory progenitor cells revert to stem cells upon crypt damage. Nature Cell Biol.

[b7] Buczacki SJ, Zecchini HI, Nicholson AM (2013). Intestinal label-retaining cells are secretory precursors expressing Lgr5. Nature.

[b8] Novelli M, Cossu A, Oukrif D (2003). X-inactivation patch size in human female tissue confounds the assessment of tumor clonality. Proc Natl Acad Sci U S A.

[b9] Fuller CE, Davies RP, Williams GT (1990). Crypt restricted heterogeneity of goblet cell mucus glycoprotein in histologically normal human colonic mucosa: a potential marker of somatic mutation. Br J Cancer.

[b10] Novelli MR, Williamson JA, Tomlinson IP (1996). Polyclonal origin of colonic adenomas in an XO/XY patient with FAP. Science.

[b11] Taylor RW, Barron MJ, Borthwick GM (2003). Mitochondrial DNA mutations in human colonic crypt stem cells. J Clin Invest.

[b12] Greaves L, Preston S, Tadrous P (2006). Mitochondrial DNA mutations are established in human colonic stem cells, and mutated clones expand by crypt fission. Proc Natl Acad Sci U S A.

[b13] McDonald S, Greaves L, Gutierrez-Gonzalez L (2008). Mechanisms of field cancerization in the human stomach: the expansion and spread of mutated gastric stem cells. Gastroenterology.

[b14] Nicholson AM, Graham TA, Simpson A (2012). Barrett’s metaplasia glands are clonal, contain multiple stem cells and share a common squamous progenitor. Gut.

[b15] McDonald S, Preston S, Greaves L (2006). Clonal expansion in the human gut: mitochondrial DNA mutations show us the way. Cell Cycle.

[b16] Greaves LC, Elson JL, Nooteboom M (2012). Comparison of mitochondrial mutation spectra in ageing human colonic epithelium and disease: absence of evidence for purifying selection in somatic mitochondrial DNA point mutations. PLoS Genet.

[b17] Baker AM, Cereser B, Melton S (2014). Quantification of crypt and stem cell evolution in the normal and neoplastic human colon. Cell Rep.

[b18] Jones S, Chen W, Parmigiani G (2008). Comparative lesion sequencing provides insights into tumor evolution. Proc Natl Acad Sci U S A.

[b19] Potten CS, Kellett M, Rew DA (1992). Proliferation in human gastrointestinal epithelium using bromodeoxyuridine *in vivo*: data for different sites, proximity to a tumour, and polyposis coli. Gut.

[b20] Potten CS, Loeffler M (1990). Stem cells: attributes, cycles, spirals, pitfalls and uncertainties. Lessons for and from the crypt. Development.

[b21] Westphalen CB, Asfaha S, Hayakawa Y (2014). Long-lived intestinal tuft cells serve as colon cancer-initiating cells. J Clin Invest.

[b22] Barker N, Ridgway R, van Es J (2009). Crypt stem cells as the cells-of-origin of intestinal cancer. Nature.

[b23] Sangiorgi E, Capecchi MR (2008). Bmi1 is expressed *in vivo* in intestinal stem cells. Nature Genet.

[b24] Schwitalla S, Fingerle AA, Cammareri P (2013). Intestinal tumorigenesis initiated by dedifferentiation and acquisition of stem-cell-like properties. Cell.

